# Developing a community-prioritized research agenda on primary care strategies for minimizing the health impacts of heat and poor air quality events: protocol

**DOI:** 10.3389/fclim.2025.1703757

**Published:** 2026-01-15

**Authors:** Rachel Gold, Hossein Estiri, Arwen Bunce, Brenda M. McGrath, Rose Gunn, Anna Steeves-Reece, Chirag J. Patel, Maura Pisciotta, Katie George, Karen Albright

**Affiliations:** 1 OCHIN, Inc., Portland, OR, United States; 2 Department of Medicine, Harvard Medical School, Boston, MA, United States; 3 Department of Biomedical Informatics, Harvard Medical School, Boston, MA, United States

**Keywords:** community health centers, extreme heat, extreme poor air quality, primary care, protocol

## Abstract

**Introduction:**

Extreme heat / poor air quality events can adversely impact health, and low-income communities are especially vulnerable to these impacts. Evidence-based interventions that minimize these impacts are needed and ideally will be developed and led by front-line organizations such as primary care community health centers (CHCs). A community-driven and -prioritized research agenda is necessary to guide the generation of evidence that CHCs need to intervene effectively. We will generate this agenda via the following protocol.

**Methods:**

We will first describe patterns of heat- and air quality-induced health impacts in a national CHC network (>2,400 clinics), focusing on hypertension and asthma. Results will be shared with advisors including: CHC staff and patients; representatives of community organizations focused on heat / air quality and health; and scientists with expertise in relevant fields. Advisors will identify additional needed analyses and consider potential interventions for interrupting the health impacts reflected in analysis results. Realist-informed qualitative data will be collected to identify likely mechanisms of effects of possible interventions. Using these inputs, we will identify a set of potential CHC-led interventions which will be categorized using a structured framework and then prioritized in terms of needed evidence on effectiveness, acceptability, and other factors. Several conceptual models guide this protocol.

**Results:**

This study began in Fall 2024, funded by the National Institutes of Health as a center planning grant. Quantitative analyses and qualitative data collection are underway, to be completed by Spring 2026; advisory committees will meet continuously through September 2027 to provide feedback, request additional analyses, generate potential interventions, and identify evidence needs and a related prioritized research agenda.

**Discussion:**

CHCs need evidence on: heat and air quality-induced health impacts in the populations they serve; how to intervene to minimize these impacts; and how to implement and sustain such interventions. Little prior research has identified or tested the effectiveness of such interventions in these settings. The process described here will create a research agenda on intervention effectiveness that reflects CHCs’ needs. It will be the first research agenda developed in partnership with CHC staff and patients, community organizations, and multidisciplinary scientists in the United States.

## Introduction

Extreme heat events and poor air quality events are increasingly common (Program., U.S.G.C.R, 2018; Monier and Gao, 2015). Both can impact health; population-level associations have been established between exposure to extreme heat and / or poor air quality and multiple adverse health outcomes (Chang, 2022). Low-income communities are the most vulnerable to these impacts because they are often located in areas with heightened vulnerability to these events (e.g., with few trees and poor baseline air quality due to proximity to high-traffic roadways and / or industrial pollution) and lack resources for responding to these events (Chang, 2022; Anderson and Bell, 2011; Astrom et al., 2011; Bell, 2018; Bunker et al., 2016; Mannucci and Ancona, 2021; Song, 2017; Sanidas, 2017; Zhang et al., 2025; Keleb et al., 2025). In the United States, such communities already have high rates of poor health outcomes (McDermott-Levy et al., 2021; Renteria, 2022; Tee Lewis, 2023). This is largely driven by exposure to adverse area-level contextual drivers of health (e.g., inadequate public transportation or affordable housing) which cause individual-level *social risks* (e.g., housing and transportation insecurity) that hamper individuals’ ability to engage in health-supporting behaviors. Social risks also limit individuals’ ability to minimize the health impacts of extreme heat / poor air quality events; e.g., housing and transportation quality determine the risk of exposure to heat and unhealthy air (Bell, 2018; Lehnert et al., 2020; Levy and Patz, 2015).

Evidence-based interventions are needed to help low-income communities prepare for and respond to these health impacts (Ghosh et al., 2022; Temte, 2023; Hess, 2023). To optimize their potential effects, such interventions must be developed and led by trusted community-based organizations. To that end, the National Academies has called for “research in partnership with affected communities to generate evidence on effective clinic-led interventions / solutions” (National Academies of Sciences, E. and Medicine, 2022). In many low-income communities, community health centers (CHCs) such as federally qualified health centers and rural health centers are key sources of healthcare access as they provide primary care regardless of patients’ ability to pay, have experience in addressing social risks, and are positioned to respond to emergent community needs (Chartbook, 2023; Health Resources & Services Administration, 2023; Adashi et al., 2010; Cole, 2021; Lewis et al., 2021; Kadandale et al., 2020; Duns, 2020). An estimated 1,370 CHC organizations (»15,000 clinics) in the United States annually provide primary care to 31 million members of low-income communities. This includes: one in 11 Americans; one in six Medicaid beneficiaries; one in five uninsured persons; and one in three persons living below the federal poverty level (FPL; Chartbook, 2024). Forty percent of CHCs are in rural areas (Pillai and Tolbert, 2025).

As CHCs are the front line of managing extreme heat / poor air quality-induced health impacts in the communities most vulnerable to these impacts (Seehusen, 2024; Hertenstein Perez, 2024), they are ideally positioned to intervene to prepare for and minimize these impacts. In fact, section 330 of the Public Health Service Act authorizes CHCs to provide services to address the health impacts of factors such as heat and air quality exposure (Health Resources & Services Administration, 2019). Such efforts are already underway in some CHCs. For example, some CHCs conduct needs assessments to identify potential impacts of heat / air quality events on patient care, and use these assessments to guide plans for mitigating these impacts (Juarez, 2025). As extreme events can interrupt CHC operations, (Wiskel et al., 2024) some CHCs are enhancing their resiliency by installing backup systems to ensure continuous care during power grid disruptions (CR4HC, 2024; Dempsey and Weiss, 2024; Burkholz, 2024). Other CHCs are partnering with community organizations to create “resiliency hubs” (Dempsey, 2023; Umpierre, 2024; Robison, 2018) by developing plans to expand mental health services and / or resource navigation support after extreme heat / poor air quality events (Galván, 2018; Seda, 2019). Others target patients assumed to be at elevated risk from heat / air quality events, such as those with hypertension and / or without stable housing (Seda, 2018), using patient education and / or proactive outreach (Wiskel et al., 2024; Hassan et al., 2024).

Yet while these varied efforts are ongoing in some CHCs, they have not been formally tested; we know of no formal intervention effectiveness testing in United States CHC settings. Prior research on primary care-led interventions targeting the health impacts of extreme heat / poor air quality events was conducted in countries with primary care systems that differ substantially from that in the United States (Blashki et al., 2007; Crandon, 2022; Watson et al., 2023; Bailie, 2023; Blane and Basu, 2023; McMichael, 2013; Ossebaard and Lachman, 2021). As such events become more frequent, however, CHCs in the United States will need empirical evidence on which interventions are most effective at minimizing their impacts. Research is needed to address this lack of evidence (Ghosh et al., 2022; Temte, 2023; Watson et al., 2023; Madrigano et al., 2021; Xie, 2021). To maximize its utility, this research must be guided by community-driven and -prioritized needs. This paper describes the protocol that will be followed to generate a research agenda on CHC-led interventions to minimize the adverse health outcomes of extreme heat / poor air quality events. This effort is the central work of the NIH-funded *Community CATALYST* planning grant. The agenda created through this process will be CHC-directed and informed by current scientific evidence on primary care-led interventions, intervention implementation, and the structural / contextual factors that support or hinder such efforts.

## Methods and analysis

### Setting

This protocol will be conducted at OCHIN, a health informatics non-profit that serves a national network of CHCs. All OCHIN member CHCs (federally qualified health centers, look-alikes, rural health clinics, local health departments, etc.) share a single centrally-managed instance of the Epic electronic health record (EHR), which includes a range of primary care data for >9.7 million patients served by >2,400 clinics located across 40 states.

### Advisors

As described below, all steps will involve three advisory committees convened for this purpose. These include: (1) 10 representatives (staff and patients) from five CHCs recruited from OCHIN’s national network, purposively sampled to ensure variability in location and population served; (2) eight scientists with expertise in relevant domains; and (3) 12 leaders of non-profit organizations focused on policy related to extreme heat / poor air quality and health at the community, state, and national levels. Each committee will meet three times per year; individually in the first year, jointly in the second year, and collectively in the third year.

### Conceptual models

Several conceptual models underlie this protocol.

The Assessing Community Engagement Conceptual Model (Aguilar-Gaxiola, 2022) underlies the protocol’s focus on engaging partners from impacted communities to guide the development of this research agenda. By centering community engagement and core engagement principles, this model informs all community engagement processes described below.

The US Global Change Research Program model shows that extreme temperature events and poor air quality events have especially harmful health impacts among persons with social risks (Kynett and U.S. Global Change Research Program [USGCRP], 2025). This underlies the need for research on interventions in the CHC setting specifically, as rates of social risks are high in CHC-served populations (USGCRP, 2016). This model also shows that identifying potential effect modifiers is essential to developing CHC-led interventions because multi-level factors determine vulnerability to these health outcomes in individuals and communities.

The Consolidated Framework for Implementation Research (CFIR; Damschroder et al., 2022) outlines potential barriers to implementing interventions in clinical settings. Such barriers can relate to the intervention itself, characteristics of the inner (e.g., clinic-level factors) and outer settings (e.g., local / national policies) and the individuals involved in the intervention, and the specifics of how the intervention was implemented. The CFIR will inform consideration of the barriers CHCs might face and associated strategies they might use to implement proven interventions, to ensure that interventions identified for future testing in the CHC-prioritized research agenda are designed with dissemination in mind.

The ‘Five As’ of social risk integration described in a 2019 National Academies Report (NASEM, 2019) categorize clinic interventions for mitigating social risks’ health impacts. The Five As—Awareness, Assistance, Adjustment, Alignment, and Advisement (or Advocacy) will guide the identification of potential CHC interventions because social risks impede resilience to extreme heat / poor air quality events. For example, persons lacking reliable transportation or housing are at greater risk of exposure to heat and poor air quality; financially insecure persons may be unable to afford air conditioners and purifiers, or the electricity needed to use them. Therefore, many CHC strategies for alleviating extreme heat- / air quality-induced health impacts will involve addressing social risks, and others will include applying the 5 As approaches to minimize these impacts. Use of the Five As is intended to ensure that known strategies for mitigating contextual impacts are the foundation for identifying interventions related to extreme heat / poor air quality events, because strategies that CHCs may use to mitigate heat- / poor air quality-induced health impacts fall into the same categories. Table 1 gives examples of potential CHC-led interventions designed to minimize the health impacts of extreme heat / poor air quality events by addressing contextual drivers of health.

### Research agenda development process

Generating a community-prioritized research agenda on CHC-led interventions to address extreme heat / poor air quality event-induced health impacts requires first understanding these impacts in CHC populations. Evidence shows that extreme heat / poor air quality events impact health, especially in low-income communities. However, there are key limitations to most prior research in this area because that research predominantly used claims-based data, which excludes uninsured persons—i.e., those served by CHCs. As a result, little is known about these impacts in geographically variable CHC populations. Second, little is known about the effect modifiers of such impacts; identifying these could help to identify causal pathways in these populations, which could illuminate likely interventions. Such effect modifiers might include social risks like housing, transportation, utilities insecurity, or occupational exposures, all highly prevalent in CHC populations, and / or community characteristics (e.g., rural vs. urban location; Bell, 2018; Ghosh et al., 2022; Temte, 2023; Bailie, 2023; Dewi et al., 2023; Applebaum et al., 2016). Addressing these evidence gaps is the necessary foundation for identifying potential clinic-led interventions for effectiveness testing in CHC communities.

**Figure 1:**
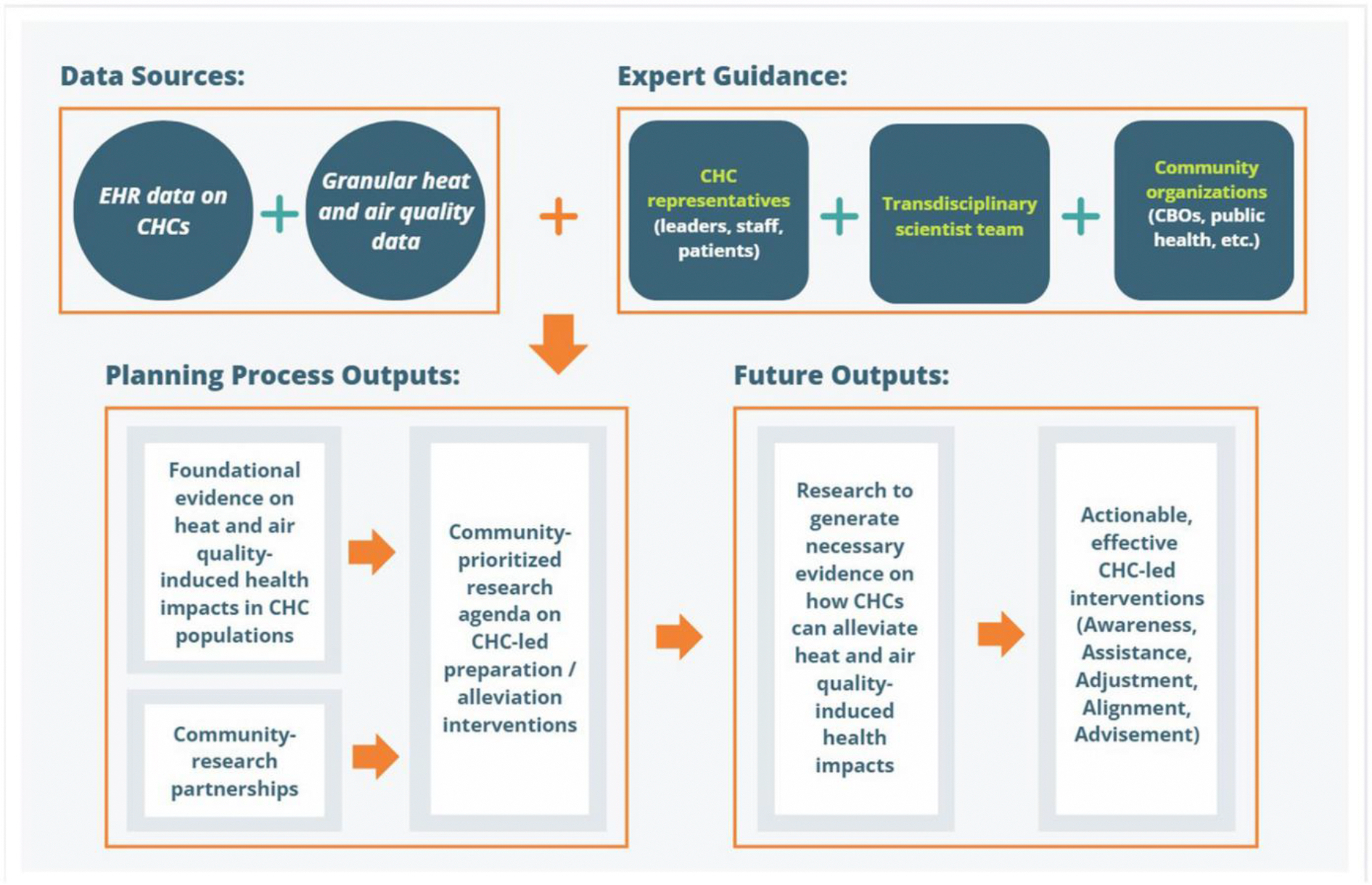
Overview of the research agenda development process

To that end, this research agenda development protocol starts by describing patterns of extreme heat / poor air quality event-induced health impacts in CHC populations, with a focus on hypertension and asthma incidence and exacerbation. Concurrently, realist-informed qualitative research will identify the mechanisms or pathways likely to effectively address the health impacts of extreme heat / poor air quality events across different CHC contexts. (A realist approach is an iterative process of developing, testing, and refining program theories to uncover mechanisms between actions and outcomes; Pawson, 1997; Westhorp, 2014). Figure 1 summarizes the proposed work: data from the EHR shared by the OCHIN national CHC network will be linked to 10 years of data on extreme heat / poor air quality events. The dataset thus created will be analyzed to generate foundational evidence on how extreme heat and poor air quality impact health outcomes in CHC patients.

The health impact patterns described in the quantitative analyses and the mechanisms identified in the realist analyses will be shared with the project advisors through a structured process designed to identify and prioritize what evidence CHCs need to effectively minimize the adverse health impacts of extreme heat / poor air quality events on the populations they serve. The advisors will use these findings to construct potential interventions intended to alleviate the negative impacts of extreme heat / poor air quality on patients and communities. They will also identify additional analyses needed to focus future intervention research, and those analyses will be conducted as feasible. All potential interventions will then be categorized using a structured framework, which will be used to support identifying which potential interventions are most prioritized by the advisors in terms of needed evidence on effectiveness and other elements. Using interactive convergent mixed methods (Fetters et al., 2013), quantitative and qualitative results will inform and build on each other in iterative cycles of data collection and analysis. The details of the steps involved in this process are described below.

This study was funded by the National Institutes of Health in September 2024 as a P20 center planning grant. Study activities began in Fall 2024. Steps 1 and 2 will be completed by late 2025, Step 3 in Fall 2026, Step 4 by late 2026, and Step 5 by Spring 2027. Results will be disseminated in Summer 2027; expected outputs include peer-reviewed papers and presentations on all analysis results, and the resulting research agenda. The Advarra Institutional Review Board reviewed and approved the study in August 2024.

### Step 1. Quantitatively describe patterns of the impacts of extreme heat / poor air quality events on hypertension and asthma outcomes in CHC populations.

Data on health outcomes will come from the OCHIN Epic electronic health record (EHR) system. As of June 2025, >9.7 million patients were in the OCHIN network’s EHR data; most live below 200% of the federal poverty line, 49% are insured through Medicaid, 25% are uninsured, and 22% speak Spanish. An estimated 21% of adults have hypertension, and an estimated 9% of adults and 7% of pediatric patients have asthma.

Data on extreme heat / poor air quality events come from Harvard’s Confluence Project (The Confluence Project, 2025), which developed a dataset on extreme temperature events in the contiguous United States since 2008, by hour, at the census block level; data from 2013 to 2023 will be used here (Fard et al., 2025). Data on poor air quality will come from a latitude / longitude spatiotemporal database of air quality events in the United States.

We will link the EHR data with temperature extremes and air quality data using geocodes. Details on the methods of this linkage process will be presented in a separate manuscript (in development). This linked EHR-heat and -poor air quality dataset will be analyzed to describe patterns of the health impacts of such events in CHC populations. We will focus on the relationship between exposure to extreme events and risk of blood pressure (BP) elevation and hypertension incidence and exacerbation among adult CHC patients, and risk of asthma exacerbation and incidence in adult and pediatric CHC patients. All occurrences of patients in OCHIN member CHCs being exposed to extreme heat events in 2013–2023 will be identified, and then those exposed to air quality events. Analyses will then determine the extent to which persons exposed to such events have a higher incidence of the outcomes of interest.

In analyses of the impact of extreme heat / poor air quality event exposure on BP, the cohort will be adults with at least one healthcare visit with a BP reading during the study period, 2013–2023. Patients will be classified based on their BP status in the year before a given visit. Analyses will use generalized linear mixed models to assess the independent associations (main effects) of acute and recurrent exposure to extreme heat / poor air quality exposure on BP (continuous measures of both systolic and diastolic BP, conducted separately). We will also examine the joint association (i.e., interaction) between extreme heat and poor air quality exposure. Random effects will be included to account for repeated healthcare encounters of individuals over time and for the nesting of patients within clinics. We will adjust for potential confounding factors in the models, including patient age, sex, insurance status, comorbid conditions, Federal Poverty Level, etc. We will account for the presence of various illnesses associated with decreased BP. We will also conduct subgroup analyses to determine whether patients with different hypertension statuses experience varying degrees of BP impact post-exposure.

We will assess the robustness of our findings and address potential biases by conducting sensitivity analyses to minimize selection bias due to the non-random assignment of treatments. Exposure to extreme heat / poor air quality is tied to geographical location, which could introduce bias if factors influence both the likelihood of living in an area with extreme heat / poor air quality and hypertension outcomes. By using matching weights, we aim to mitigate this bias by re-weighting the study population to create a hypothetical balanced sample where treatment assignment (i.e., exposure to extreme heat or poor air quality) is independent of potential confounders. We will weight the two groups based on multiple patient characteristics. The analyses will then be repeated to assess impacts on asthma exacerbation in which the cohort will be children or adults with at least one healthcare visit during the study period. Details of the planned analyses will be presented in a separate manuscript (in development).

### Step 2. Identify effective mechanisms of impact to undergird future interventions, using a realist-informed approach.

Developing successful CHC-led interventions targeting the health impacts of extreme heat / poor air quality events requires pinpointing the underlying mechanisms through which such interventions may be most effective. We will use a realist-informed approach to identify these context-driven mechanisms. First, we will conduct a modified rapid realist review to identify potentially effective mechanisms (often tacit theories or evidence about what worked and why in similar situations) from the peer-reviewed and gray literature (Saul, 2013; Rycroft-Malone, 2012; Flynn, 2020; Groot et al., 2017). In the context of CHCs, examples of relevant mechanisms may include perceptions related to patient-care team communication. The potential mechanisms identified in the review will then guide subsequent primary data collection. During that data collection we will look for—and explicitly ask about—evidence that supports or challenges the hypothesized potential of these mechanisms to reduce the health effects of extreme heat / poor air quality in populations served by CHCs (details below).

To collect those data, we will conduct one in-person site visit at each of the five CHCs advising the project, at which we will conduct a patient focus group, three staff interviews, and observations of clinic workflows and the structure and culture of the organizational environment. Staff will be selected to participate in interviews based on their interest in and experience addressing related health issues. The site visits will be structured to gain granular understanding of relevant needs, constraints, and opportunities at each CHC and to investigate (per the realist approach) how, for whom, and under what circumstances CHCs can act to alleviate extreme heat- / poor air quality-induced health impacts in their patients and communities. As noted above, we will focus primarily on the mechanisms identified in the realist review, including contextual factors that may impact each mechanism’s effectiveness, while remaining open to emerging concepts and ideas. Concurrently, we will conduct in-depth virtual interviews with patients and staff (*n* = 10) and leaders of community organizations (*n* = 12) on our advisory committees. Similar to the site visit data collection, these interviews will be exploratory and focus on potential mechanisms of impact. Learnings from the observations, interviews, and focus groups will be integrated into committee advisory meeting discussions to inform development of the research agenda on potential interventions. All advisory committee meetings will be observed and resulting fieldnotes included in analysis.

To identify potential interventions and the mechanisms through which they may have impact, site visit data collection will be focused as follows. We will investigate the personal experiences, knowledge, and perspectives of CHC staff as they relate to such mechanisms, notably their views on the role of the clinic in addressing related health impacts, the relationship between the clinic and the community it serves, and ideas about possible interventions and experience with any relevant programs currently in place. We will review the clinics’ disaster and emergency management plans, a rich source of information on how CHCs position themselves and their communities for resilience following disasters. We will document other contextual factors including clinic workflows and the structure and culture of the organizational environment. We will query patients about their lived experience with respect to extreme heat / air quality events and their effects on health, their experiences at the clinic, their reflections on mechanisms identified through the realist review such as psychological safety, control / empowerment, and shared experience, and their perceptions of whether the clinic has a role to play in supporting community resiliency and, if so, what that role should be (addressing issues of respect and power dynamics as well as pragmatic ideas for support and intervention).

Data collection and analysis will be composed of iterative cycles of theory generation, testing, and refinement. We will integrate grounded theory and the realist approach in coding and analysis to preserve the richness and nuance of the data while moving toward causal theories with explanatory power. The initial phase of analysis will follow an open coding technique in which concepts grounded in the empirical data are labeled (coded) and grouped into categories. The second phase will begin to make explicit the connections between concepts and categories based on the realist ontology, which combines empirical observation, pre-existing theory, and insights from the literature to identify underlying generative mechanisms and contingent conditions (context) to produce explanations with causal depth.

Finally, mixed methods analyses will use a convergent comparative case analysis approach in which quantitative and qualitative results are integrated to inform the development of theories regarding how CHC-led interventions may offset the health effects of extreme heat / poor air quality events, to guide selection of intervention priorities. This integration will provide understanding of how CHCs may most effectively mitigate these health impacts in the populations they serve.

### Step 3. Advisors identify potential interventions based on Step 1–2 results.

Concurrent to Steps 1–2, the advisory committees will discuss perceptions and concerns about how extreme heat / poor air quality events impact CHC populations and potential constraints and opportunities for CHC-led interventions to minimize these impacts. Emergent data on health impact patterns from Step 1 and anticipated mechanisms of intervention from Step 2 will be shared with advisors for collaborative interpretation.

Based on these findings, the advisors will identify an initial set of potential CHC-led interventions for minimizing the impacts of extreme heat / poor air quality events, as follows. In addition to emergent results from Step 1–2 analyses, the advisors will also review overviews of: the current evidence on the effectiveness of relevant clinic-led interventions targeting social risks’ health impacts, per the ‘Five As’; the current evidence on CHC-led interventions proven effective at supporting chronic disease management in general; potential interventions specific to the impacts of extreme heat / poor air quality events that have been studied in countries other than the United States; and those that have been conducted in other United States CHCs without formal study, as found in the gray literature and online. The advisors will then be asked to identify which interventions from these sources are potentially of interest for interrupting the identified impacts of extreme heat / poor air quality events in CHCs via specific identified mechanisms. Their responses will comprise the initial list of interventions for prioritization.

The advisors will also determine what additional quantitative, qualitative, or other evidence is needed to refine that set of interventions. These will determine the focus of the Step 4 analyses; given resource constraints, they will be prioritized by consensus. Questions requiring qualitative data will inform what is covered in the second round of qualitative data collection, and those requiring quantitative data will inform the content of subsequent quantitative analyses. To ensure that the Step 4 quantitative analyses involve identifying effect modifiers that influence the causal pathways through which extreme heat / poor air quality events may have differential impacts on different groups, the advisors will be asked to identify and prioritize potential effect modifiers for evaluation. Needed evidence that cannot be generated via this process will be noted for future consideration in the research agenda developed here.

### Step 4. Provide requested data as feasible; refine understanding of potential interventions.

A second round of analyses will then be conducted to provide answers to the questions that emerged from study advisors in the prior step. These analyses will also be structured to synthesize the findings of this process as follows.

To collect any qualitative data identified as needed, we will conduct a second (virtual) patient focus group, as possible with the same individuals that participated in the prior discussion, and two virtual interviews with staff in leadership positions at each of the five participating CHCs. These interviews and focus groups will focus on participants’ insights into the patterns identified in the quantitative work, reactions to recommendations from the advisory committees, and brainstorming / refining potential interventions and implementation strategies based on the mechanisms prioritized in Step 2.

The last set of qualitative analyses will focus on refining our understanding of the potential causal links (mechanisms) between actions and outcomes most likely to motivate individuals and communities to act in ways that alleviate the health impacts of extreme heat / poor air quality events, and on contextual factors that facilitate or impede activation of these possible mechanisms in varied situations. Data from all prior observations, patient focus groups, staff and advisor interviews, the content of advisory committee discussions, and the results of the quantitative analyses of extreme heat / poor air quality and EHR data will be merged to build an understanding of what works, for whom, in what circumstances. This insight will guide intervention selection, structure, and prioritization.

The last set of quantitative analyses will focus on identifying potential causal pathways (effect modifiers) influencing the patterns of extreme heat / poor air quality-induced health impacts in CHC populations. They will be structured to determine how factors such as geographic region, urban / rural location, and patient sociodemographic characteristics including self-reported social risks are associated with differences in the risks of extreme heat- / poor air quality-induced health outcomes. As there may be multiple drivers of such variation at multiple levels of influence, we will conduct analyses similar to those in Step 1 but including interaction terms incrementally to evaluate how event exposure impacts vary across distinct subgroups. The results from these mixed methods analyses will demonstrate how the effects of extreme heat / poor air quality events vary among CHC patient subgroups. This information will directly inform the process of intervention categorization and prioritization.

Table 2 gives examples of research on potential interventions that might be identified as needed.

### Step 5. Potential interventions categorized and prioritized.

Emergent Step 4 results will be shared with the project advisors to help them refine a list of potential interventions for which effectiveness research is needed. To structure that process, a set of questions will be answered for all potential interventions to support obtaining stakeholder input on the most important research to pursue in future studies. The planned questions are listed in Table 3 with an example of how they might be answered; questions will be added to this list if indicated in prior steps.

For each potential intervention, the mechanism of expected impact will be determined to ensure that this is explicit and aligned with realist evaluation findings. A high-level summary of prior evidence on a given intervention will be included to help focus on what evidence is most needed overall and for patients in different age groups. Which of the ‘Five As’ is relevant will be included because many potential interventions will likely directly address social risks and / or will be addressed using strategies similar to those now used by CHCs to address such risks. The questions will also include details on what evidence is needed for a given intervention—effectiveness alone, or feasibility and acceptability? The latter two will be relevant to understanding the evidence required to ensure that effective interventions are sustainably implemented and widely scaled, as will the questions on whether implementation barriers might hamper such actions (per CFIR categories) and strategies to address them. This emphasis on implementing, spreading, and sustaining interventions is critical as implementing new processes in clinics necessitates ‘implementation strategies’ (Powell, 2015; Waltz, 2015) such as practice coaching, training, and financial incentives. These are known to be differentially effective at supporting uptake of varied interventions in different contexts, so supporting practice change requires identifying which may work in a given setting.

Once each potential intervention has been defined per these standardized questions, each will be iteratively prioritized. Using a web-based prioritization matrix (Neudorf et al., 2015; North and Varkey, 2010), we will ask all study advisors to give input on the most critically needed evidence. Each advisor will have several opportunities to ‘vote’ for interventions for which effectiveness evidence is needed and whether there are specific questions of more or less importance related to that intervention. The final list of research priorities thus generated will be a community-generated agenda for future research on CHC interventions to mitigate patterns of extreme heat / poor air quality-induced health impacts, and the support CHCs need to sustainably implement proven interventions.

### Progress to date

As of December 2025, we have completed the following pieces of the protocol. Within Step 1, we have: created the linked heat events—EHR dataset; identified persons with hypertension in the dataset; almost completed creating the linked air quality events—EHR dataset (data now linked for 2019–2023); and conducted analyses of associations between exposure to heat events and blood pressure (results currently being prepared for peer review). Within Step 2, we have: conducted a modified rapid realist review to identify potentially effective mechanisms from current literature, to inform subsequent data collection; conducted four of five site visits, with data currently being analyzed; and conducted multiple advisory group meetings at which we collected Step 2 data as described above. One notable challenge involved the complexity of linking individual patient address data to heat event data; we resolved this by assigning patients’ heat exposure to their clinic’s address, on the assumption that local variation in heat exposure would be minimal.

## Discussion

In the United States, CHCs increasingly need interventional strategies to prepare for and minimize extreme heat- / poor air quality-induced health impacts in the communities they serve. While many CHCs already seek to intervene to minimize these health impacts on their patients, they lack empirical evidence on the effectiveness of such efforts. Most prior research on primary care-led interventions targeting these health impacts was conducted in countries other than the United States, whose primary care systems and social support structures differ substantially from those in the United States (Blashki et al., 2007; Crandon, 2022; Watson et al., 2023; Bailie, 2023; Blane and Basu, 2023; McMichael, 2013; Ossebaard and Lachman, 2021). Given the many potential strategies that United States CHCs might use to address these health impacts, those they implement must have demonstrated acceptability and effectiveness in their populations and settings. CHCs therefore need evidence on heat- / poor air quality-induced health impacts in their populations, how to intervene with optimal impact, and how to implement and sustain such interventions. There is a clear need for an applied research agenda on intervention effectiveness that reflects the needs of CHCs.

The planning process described here will create that agenda by identifying the research priorities of CHCs and their communities; it will be the first research agenda in the United States on primary care interventions to minimize extreme heat / poor air quality-induced health impacts developed in partnership with CHC-based advisors, community organizations, and scientists. The research agenda thus created will be widely disseminated through multiple routes. In addition, the data infrastructure created here will be a unique resource for researchers interested in studying interventions that address such health impacts in CHC populations.

There are some limitations to this protocol. First, heat and air quality impact health outcomes managed in primary care settings beyond those of focus here (e.g., mental health; [Saeed and Gargano, 2022; Siskind et al., 2016]; maternal and child health [Roos, 2021; Roos et al., 2021; Rylander et al., 2013]). While our advisors may prioritize some of these factors, resource limitations apply and future efforts may need to expand on the agenda created here. Nevertheless, heat and poor air quality events are rapidly increasing, and hypertension and asthma are prevalent in CHC populations, making this an appropriate starting place. Similarly, the EHR data used here comes from ambulatory primary care settings, so outcomes involving other settings such as emergency departments are not captured. Finally, it is reasonable to ask the extent to which CHCs should be responsible for addressing extreme heat / poor air quality-induced health impacts. We posit that CHCs’ commitment to the health of the communities they serve make such intervention necessary as increasingly frequent extreme heat and poor air quality events will disproportionately impact the health of their patients, whose high rates of social risks heighten their vulnerability to these impacts. Furthermore, as noted above, the Public Health Service Act recognizes that addressing factors such as extreme heat / poor air quality is within the scope of CHCs’ mission.

## Figures and Tables

**Table 1:** Examples of potential CHC interventions aligned with the Five As of Social Risk Integration

Five As	Potential clinic-led interventions
**Awareness:** *Ensure care team knowledge of conditions caused or exacerbated by extreme heat / poor air quality events*	Present actionable data in the EHR to let care teams know when certain patients are at risk for extreme heat / poor air quality-induced health impacts, e.g., heat or air quality alerts for patients with hypertension or asthma who have transportation insecurity
	Conduct screening to identify patients who lack air conditioners / purifiers, or are unable to use such devices due to increased utilities cost; provide information on local resources to address such needs
	Build community awareness about specific extreme heat / poor air quality-induced health risks, e.g., educating community members about how to prepare for / respond to risk-increasing events
**Assistance:** *Refer patients to resources to minimize extreme heat / poor air quality health impacts*	Provide referrals to community services / local resources / public health departments that may address social risks with the potential to exacerbate extreme heat / poor air quality-induced health impacts
	Provide air purifiers / conditioners, or referrals to community resources for obtaining them
	Provide or refer for transportation / food or for utilities payment assistance programs
	Provide information and/or facilitate access to community locations providing shelter (including cooling, heating, and/or filtered air) during extreme heat / poor air quality events
**Adjustment:** *Change care plans as needed*	Provide mental health services to address psychological distress associated with extreme heat / poor air quality events
	Provide care elements via telehealth when heat / air quality / other extreme events make travel dangerous
	Ensure medications are mailed to the patient when heat / air quality / other extreme events make travel dangerous
**Alignment:** *Clinics partner / collaborate with other community organizations*	Partner with local public health departments to provide information on evacuation procedures, shelter locations, access to medical care, and health risks
	Identify populations at high risk of extreme heat / poor air quality impacts, e.g., with pre-existing conditions, in collaboration with public health agencies / other local organizations
	Develop emergency response plans with local emergency management agencies to ensure CHCs are able to provide care during and after extreme heat / poor air quality events; includes contingency plans for power outages, increased patient load
**Advisement:** *Provide needed data to support data-driven policies*	Generate data that can be used to inform local / national policies related to reimbursement for needed services (e.g., healthcare and public health disaster preparedness)
	Generate localized data on extreme heat / poor air quality-induced health impacts to inform local and national policies*
	Document health impacts of extreme heat / poor air quality events; identify communities with high vulnerability to these impacts, to prioritize strategies that build their resilience*

* Intervention / action recommended explicitly by the National Academies 2022 report[80]

**Table 2:** Examples of research that might be conducted to meet evidence needs identified here

**Evaluate Awareness interventions**	What information in the EHR helps CHC staff identify patients whose health is likely to be impacted by extreme heat / poor air quality events? What form should it take (outreach registries, clinical decision support, etc.)? What staffing / reimbursement models support effective use of such data? What EHR-based tools support CHC extreme heat / poor air quality event response / adaptation / resilience interventions (e.g., registries, alerts, bulk communication, etc.)?
	How can CHCs best communicate with their patients about extreme heat / poor air quality event-induced health risks (e.g., air quality alerts targeting patients with specific morbidities)? Use of patient portal, bulk texting?
**Evaluate Assistance interventions**	What strategies ensure that patients have resources to be resilient to extreme heat / poor air quality events? What resources can CHCs provide directly? What clinic processes seamlessly connect patients to community organizations that provide such resources (e.g., air conditioners / purifiers; utilities payment support; community weatherization programs)?
	How do social risks interact with extreme heat / poor air quality events? How can social risk-focused interventions mitigate this interaction?
	How can CHCs support mitigating extreme heat / poor air quality impacts on agricultural workers?
**Evaluate Adjustment interventions**	How can telehealth help maintain patient access to care during extreme heat / poor air quality events? What resources would ensure that this is available to all, e.g., broadband access? What other virtual care modalities are useful?
	How else can care plans be changed to mitigate extreme heat / poor air quality-induced health impacts? What helps ensure that medication regimens are not interrupted during extreme heat / poor air quality events?
**Evaluate Alignment interventions**	How can CHCs best build / enhance partnerships with local public health departments, primary care associations, hospitals, and community organizations? How can these partnerships mitigate extreme heat / poor air quality-induced health impacts among CHC patients? How should they be structured / supported so CHCs are equal partners in such endeavors?
**Evaluate Advisement interventions**	What CHC data can inform local and national policies? What is the most effective way to provide it? E.g., describing patterns of health, generating surveillance data on CHC populations, describing regional / local / microlevel variations in these impacts (e.g., in rural vs. urban areas)?
	What policies mitigate / exacerbate extreme heat / poor air quality-related health impacts (e.g., do limits on telehealth use impact CHCs’ readiness / mitigation capacity)?
**For all the above**	What implementation strategies enhance adoption of such interventions? What level of support do CHCs need? What form should it take (reimbursement, extension center support, coaching)?
	What are “bright spots,” i.e., CHCs that developed innovative approaches? How effective were these innovations and for which patients / which circumstances? How can they be disseminated? What adaptations support doing so?
	What best practices of privately insured systems / hospitals could be translated into primary care CHCs?
	How can CHCs support communities’ ability to prepare for, adapt to, withstand and recover from extreme heat / poor air quality events?
	What needed data are not yet collected (e.g., access to air conditioning)? What would support their collection?
	What are the needs of different clinics, regions, populations? How can interventions be modified per different needs?

**Table 3:** Hypothetical example of categorizing potential interventions

Question	Example
What is the overarching research question / pattern to be addressed?	How can CHCs reduce heat events’ impact on hypertension exacerbation among persons aged >65?
Relevant to specific patient populations?	Age >65
Potential intervention?	Text targeted patients during a heat event to provide information on how to minimize their risk
Mechanism?	Automated communication of risk and preventive actions
Prior evidence?	Yes, texts can be impactful in some contexts
Which of the 5 As?	Awareness
Effect modifiers known?	Yes, patients with household income under a certain level
*Evidence needed on:* Feasibility? Acceptability?	Acceptability to CHC staff and patients
*Evidence needed on:* Effective? In which context?	Yes, in CHC patients aged >65
*Evidence needed on:* Potential barriers to intervention implementation / sustainment? Which CFIR categories?	Yes: CFIR categories = characteristics of intervention, inner setting, targeted individuals, how implemented
*Evidence needed on:* Strategies to mitigate barriers?	Yes, TBD
